# Metals and Alloys

**Published:** 1862-05

**Authors:** B. Wood


### METALS AND ALLOYS.

BY DR. B. WOOD.

I PROPOSE to submit through the REGISTER, from time to time, as opportunity offers, some observations upon the subject of Metals and Alloys. I shall treat specially of the softer or more fusible metals and metalic combinations, such as may prove useful for casting and other purposes of the laboratory; not confining myself, however, to such as are merely of practical availability, but treating the subject so as to exhibit the nature and properties of metals in their relations to each other.

In order to facilitate description in respect to some of their principal qualities, and to represent these to the eye at a glance, I have prepared the following tables, in which several well known metals are arranged as standards of comparison. On these points some preliminary observations are offered by way of explanation.

### SCALE OF HARDNESS.

Different methods of determining the hardness of metals have been adopted, none of which appear to be altogether free from objection. The most accurate mode, by means of a graduated punch worked by lever and weight, adopted by Messrs. Calvert and Johnson, is quite tedious, and the machinery too complicated for common use.* Ordinarily the main object of examining a metal in respect to this quality

---

* The method adopted by Dr. Austen, as detailed in the *American Journal of Dental Science*, consists in dropping a hammer of a given weight upon a punch pressing the metal to be experimented upon, and measuring the distance the point penetrates in different specimens. It is liable to some objections, and the shock is such as to cleave metals that are brittle. It is probably liable to give inaccurate results, especially in the case of metals of a spongy or elastic nature. Thus a piece of spongy wood will resist the blow of an axe more effectually than another of compact structure, and really the harder of the two. Although the Doctor's experiments appear to have been conducted with the necessary precautions, the result gave zinc a higher degree of hardness than copper, which may be explained on this principle: the spongy or elastic texture of zinc causing the blow to rebound.

is, not so much to determine the hardness of its particles as indicated by the common method of scratching the surface of another with it, or *vice versa*, nor to ascertain its interstitial compactness as by penetrating its substance by means of a pointed instrument under pressure of a given weight, but rather to find out the relative amount of force it, as a mass of a determinate form, will sustain without yielding or giving way, as compared with other metals in like form and condition. That is, we wish to know its state of coherence *as a body*, subjected to the test of pressure, in comparison with similar specimens of other metals, so as to determine our choice for a particular use. A piece of grindstone will *scratch* iron, though iron is much the harder. A substance may be penetrated in the direction of cleavage, or along the plane of crystallization, with ease, when, at other points, it would resist a greater force. The same may be inferred of those metals that are crystaline, or lamellar, in structure.

Nor is it the absolute hardness of a specimen that is the object of practical concern, but simply to know which of any two or more metals, relatively considered, will, in respect to this quality, best answer the end.

The mode adopted for ascertaining the relative hardness of metals represented by the table, is simple and convenient, and affords a good practical test for common use. Specimens of the metals to be examined are cast in the form of triangular bars, measuring at the sides about a quarter of an inch. These being applied edge to edge and placed under pressure in a vice, the relative hardness of any two pieces is determined by the relative amount of indentation received by them. If the one be deeply indented or cut, and the other not sensibly or but slightly notched, the former is reckoned at least one degree the harder—and if no compression be made upon it, it may claim a still higher figure in the scale; its precise place being ascertained by further comparison. If both specimens be strongly indented, although differing in degree, and the indentation of the one be no more than half that of the other, they rank in the same class: or if the difference be still greater, the one may take an intermediate place, or be ranked in the class to which it is most nearly allied, with the sign + or — attached, according to circumstances; or it may be designated by the numbers representing both classes. Thus an alloy of one part of tin and nine parts of antimony being deeply indebted by the metals in class 5, while it also reacts upon them strongly, would, to be definite, be represented by — 5. An alloy composed of lead and bismuth, each one part, and tin two parts, being nearly midway in hardness between the classes 4 and 5, may be represented by 4—5; or, for greater precision, by the use of a decimal, 4·5. These remarks apply also to the designation of other qualities.

The metals in the scale are so arranged that to this test each will be found to cut or divide the one next above it, and to be divided by that next below, without perceptible reaction in either case.

A mould for forming such bars (which, for the want of a better, could be carved out, with a chisel, from a slab of soapstone,) may be modeled so as to obtain, at the same casting, specimens for determining the qualities of flexibility and malleability, as below indicated.

FLEXIBILITY.

All metals possess some degree of flexibility. A brittle metal admits of considerable flexion when in thin plates, and the amount will depend upon the thickness of the point operated upon. By bending similar bars of different metals, at a constricted point (so as to prevent yielding at other parts), and noting the number of degrees described before breaking, we arrive at an approximation in this particular. The metals represented by the table, flexed at a point one line in thickness, breaks as follows—reckoning the angle of breakage from that at which the piece cracks or *begins* to separate:—Class 1, at 5° or less; class 2, over 5° and under 20°; class 3, over 20° and under 45°; class 4, over 45° and under 90°; class 5,

90° and upwards. A metal that may be uniformly bent the thickness named to a right angle may be accounted perfectly flexible. Beyond this the method is unsatisfactory, as the more flexible metals break gradually, sometimes thinning down to a mere thread before separating. Nor is it altogether satisfactory in other cases; and to assist our determinations, we have to take into account not only the angle of flexure, but the mode of fracture, or rather the manner of breaking, as the word "fracture" is usually applied to designate the *appearance* of broken surfaces. Some flexible metals after a certain amount of flexion break short, as though rendered brittle by the process, like cadmium; others, as tin, separate by gradual attenuation. Of the more brittle kinds, some at at a certain angle break through abruptly; others at the same point merely crack upon the surface of tensure, separating more and more as the strain is continued, and dividing completely only after a wide sweep. In short, some *tear*, some *break*, others *snap*. Again, some alloys will snap under a sudden strain that admit of considerable flexion if the force be gradually applied. A rigid description would express all these conditions, with terms appropriate to each. Moreover, in order to completeness in regard to this quality, independently considered, not only the amount of flexion, but also the force required to produce it, should be measured: but the *hardness* of the metals will afford some criterion in this respect. I have thought it not out of place, or unimportant, to thus indicate what remains to be done.

### MALLEABILITY.

The metals are classified in the scale according to the reduction in thickness they will bear under the hammer, without breaking, whether through the center or at the margin. Specimens, cast in the form of a bullet three lines in diameter, being subjected to this test, and the amount of reduction measured by means of a pair of callipers, it was found that the metals ranked in class 1 break upon being reduced less than one-tenth of this diameter (brittle); class 2 (slightly malleable) admit of being reduced from one to three-tenths (or nearly ¼ in bulk); class 3, from three to five-tenths (¼ to ½), being semi-malleable; class 4, from five to seven tenths (½ to ¾). The metals belonging to this class admit of being hammered down to thin plates, but crack more or less on the margin, and therefore, although "malleable," are not ranked perfectly so. Class 5 includes all those metals that may be beaten down nine-tenths of their thickness without breaking or cracking—the margins remaining entire—and are characterized as perfectly malleable. They may be further classified according to the usual tables.

When at a loss as to the precise place of a metal under examination, the nature of its cleavage or its subsequent "behavior" under the hammer often assists decision; or it is represented by two figures, as in the case of other qualities.

### FUSIBILITY.

The scale in the table is arranged to correspond to the actual melting point as indicated by Fahrenheit's thermometer, taking the unit to represent 100°. Class 1 includes those metals which melt at 100° and under 200°; and so of the others. Since the most careful measurements are liable to error by a few degrees, a latitude of ten degrees is allowed, (and it will be seen by reference to the table that the results of different experimenters usually vary still more widely,) so that if a substance melts at 90°, or a little upwards, it is placed in class 1; if at 190°, in class 2, &c.

It may be thought that the *mean* of two whole numbers ought to be adopted, as the unit of measure, in classification; that, for instance, metals melting at 150°, instead of 100°, should be taken as the true type of the class expressed by the unit 1, allowing a range of deviation so as to include 50° on either side. But the scale was arranged with a view to the employment, when desired, of decimals corresponding to the figures which express the absolute melting point. Thus the fusibility of a metal melting at 150° or 175° would, to be definite, be indicated by 1·5 or 1·75: while, if its precise melting point was not determined, although found by comparative tests to fall somewhere between two numbers, we should make use of both to express it; as, 1—2, or 1·5—2, which indeed, in some cases, is about as near as we can approximate the truth by actual measurement.

The table shows both the temperature of liquefaction and that of congelation, as indicated by the mercurial thermometer (Fah.), the first being assumed as the "melting point." It also exhibits in the same view, the results of different experimenters, varying somewhat from mine, but as much among themselves.

For abbreviations, I prefer using the initials of the English names of the metals, instead of the Latin.

SCALE OF HARDNESS, FLEXIBILITY, AND MALLEABILITY, WITH EXAMPLES

**HARDNESS.**

| Class. | Types. |
|---|---|
| 1. | Lead. |
| 2. | Tin.<br>1 B. 3 L. |
| 3. | 2 L. 1 T.<br>Bismuth. |
| 4. | 2 T. 1 L. |
| 5. | 1 An. 6 T.<br>Cadmium. |
| 6. | 1 An. 3 T.<br>2. An. 1 Cop. 12 T |
| 7. | Zinc. |

**FLEXIBILITY.**

| | |
|---|---|
| 1. | Bismuth.<br>1 An. 3 T.<br>2 An. 1 Cop. 12 T. |
| 2. | 6 B. 74 T. |
| 3. | Zinc.<br>8 B 5 L. 3 T.<br>1 An. 6 T. |
| 4. | 1 B. 3 L. |
| 5. | 1 A. 9 T.<br>Cadmium.<br>Lead.<br>Tin.<br>Alloys of L. and T. |

**MALLEABILITY.**

| | |
|---|---|
| 1. | *Brittle.*<br>Antimony.<br>Bismuth. |
| 2. | *Slightly Malleable.*<br>1 An. 3 T.<br>8 B. 5 L. 3 T.<br>2 An. 1 Cop. 12 T. |
| 3. | *Somi malleable.*<br>1 B. 1 L. 2 T.<br>Zinc (cast) |
| 4. | *Malleable.*<br>1 An. 6 T. |
| 5. | *Perfectly Malleable.*<br>1 B. 3 L.<br>Cadmium.<br>Lead, Tin, &c. |

(To be Continued.)

